# Simulated daylight versus conventional PDT for mild to moderate actinic keratoses: A randomized controlled trial

**DOI:** 10.1111/jdv.70556

**Published:** 2026-06-10

**Authors:** Alexandra Sjöholm, Eva Backman, Maja Modin, Julia Fougelberg, Magdalena Claeson, John Paoli

**Affiliations:** ^1^ Department of Dermatology and Venereology, Institute of Clinical Sciences Sahlgrenska Academy, University of Gothenburg Gothenburg Sweden; ^2^ Department of Dermatology and Venereology Region Västra Götaland, Sahlgrenska University Hospital Gothenburg Sweden

**Keywords:** actinic keratosis, aminolevulinic acid, photodynamic therapy, randomized controlled trial, simulated daylight

## Abstract

**Background:**

Conventional photodynamic therapy (C‐PDT) with red light is an effective but painful treatment for patients with actinic keratoses (AKs). Simulated daylight PDT (SDL‐PDT) is most likely less painful, but its effectiveness has not been thoroughly investigated.

**Objectives:**

To evaluate if SDL‐PDT is non‐inferior to C‐PDT in terms of effectiveness in the treatment of mild to moderate AKs. Additionally, pain perception, local skin reactions and patient preferences were assessed.

**Methods:**

This single‐centre, non‐inferiority (−8% margin), randomized controlled trial included adults with symmetrically distributed mild to moderate AKs across one or more anatomical sites. These sites were randomly assigned to treatment with a single session of C‐PDT on one half of the affected area and SDL‐PDT on the other half, using 5‐aminolevulinic acid hydrochloride as the prodrug. Pain during illumination was assessed using a 0–10 numeric rating scale (NRS). Patient preferences were recorded through self‐report forms. Follow‐up assessments were conducted at 3 months to evaluate early treatment failure and at 1 year to determine overall clearance rates.

**Results:**

In total, 69 participants with 1458 mild to moderate AKs were treated. Most lesions (89.9%) were located on the face and scalp and were diagnosed through clinical and dermoscopic evaluation. After 1 year, the clearance rate was 71.0% for SDL‐PDT and 85.3% for C‐PDT (*p* < 0.001), thus failing to demonstrate non‐inferiority. Illumination was reported to be more painful during C‐PDT (mean NRS score of 6.6) compared with SDL‐PDT (mean NRS score of 0.8) (*p* < 0.001). One day after treatment, 98.3% of respondents expressed a preference for SDL‐PDT.

**Conclusions:**

SDL‐PDT showed lower clearance rates than C‐PDT but caused significantly less pain. Although not supported as a primary treatment for AKs, SDL‐PDT may be considered for patients prioritizing treatment tolerability within shared decision‐making.

This randomized controlled trial was registered at http://www.researchweb.org/ (Project ID: 264721).

## Plain Language Summary

**What is the Disease?:**

Actinic keratosis (AK) is a common skin condition that produces rough, scaly patches on sun‐exposed skin. Some lesions may progress to skin cancer if left untreated.

**Location of the Study:**

Department of Dermatology and Venereology, Sahlgrenska University Hospital, Gothenburg, Sweden.

**Aim of the Study:**

To compare simulated daylight photodynamic therapy (SDL‐PDT) and conventional photodynamic therapy (C‐PDT) using 5‐aminolevulinic acid as the prodrug for treating mild to moderate AKs, focusing on effectiveness, pain, side effects and patient treatment preferences.

**Methodology:**

Sixty‐nine adults with mild to moderate AKs participated. Each patient received both SDL‐PDT and C‐PDT on separate halves of the affected area (split‐site design). Pain and local reactions were recorded during and after treatment, and lesion clearance was assessed at 3 months and 1 year.

**Key Findings:**

C‐PDT was more effective than SDL‐PDT with a 1‐year clearance rate of 85.3% compared to 71.0%. However, SDL‐PDT caused far less pain (mean NRS 0.8) than C‐PDT (mean NRS 6.6). Nearly all patients (98.3%) preferred SDL‐PDT immediately after treatment.

**Conclusions and Practical Implications:**

SDL‐PDT is less effective but also less painful than C‐PDT. The results support person‐centred treatment decisions, helping patients and clinicians balance long‐term effectiveness and comfort when choosing therapy for AK.

## Graphical abstract

This single‐centre, randomized controlled trial showed that simulated daylight photodynamic therapy (SDL‐PDT, *n* = 785 lesions) was less effective than conventional photodynamic therapy (C‐PDT, *n* = 673 lesions) in treating mild to moderate actinic keratoses with 1‐year clearance rates of 71.0% for SDL‐PDT versus 85.3% for C‐PDT (*p* < 0.001). However, SDL‐PDT was less painful (mean NRS 0.8) than C‐PDT (mean NRS 6.6) (*p* < 0.001). Despite lower effectiveness, SDL‐PDT may be considered for patients prioritizing treatment tolerability.

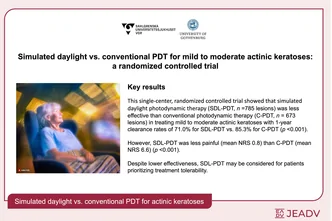

**Why was the study undertaken?**

Actinic keratosis (AK) is common and can be treated with photodynamic therapy (PDT). Conventional PDT (C‐PDT) with 5‐aminolevulinic acid (ALA) is effective but often painful. Evidence for the effectiveness of simulated daylight PDT (SDL‐PDT) using ALA is limited.

**What does this study add?**

SDL‐PDT achieves lower 1‐year lesion clearance than C‐PDT but causes significantly less pain.

**What are the implications of this study for disease understanding and/or clinical care?**

The study highlights the trade‐off between effectiveness and tolerability. C‐PDT remains more effective for complete lesion clearance while SDL‐PDT may be suitable for patients prioritizing comfort.

## INTRODUCTION

Topical photodynamic therapy (PDT) using red light, known as conventional PDT (C‐PDT), and natural daylight PDT (DL‐PDT) are both approved and widely used methods for treating actinic keratosis (AK).^1^, ^2^ Simulated daylight PDT (SDL‐PDT) is a relatively new method that most likely offers a less painful alternative to C‐PDT for treating AK without the harmful ultraviolet radiation exposure and the weather‐dependency involved in DL‐PDT.^3^, ^4^, ^5^ Unlike DL‐PDT, which requires specific daylight conditions (minimum 11,000 lux)^6^ and is dependent on weather and season,^7^ SDL‐PDT can be performed indoors in a controlled environment using simulated daylight aimed at the targeted skin area.^8^ The illuminance used in SDL‐PDT (≈12,000 lux) corresponds to an effective PpIX radiant exposure of 16 J/cm^2^ for 80 min of treatment, ensuring doses comparable to conventional PDT based on a photometric analysis described previously.^3^ However, current effectiveness data for SDL‐PDT are limited, and long‐term observations are lacking. To date, two small studies have been published on SDL‐PDT for AK, with two treatment sessions 1 week apart, using 5‐aminolevulinic acid hydrochloride (ALA). In a retrospective study involving 32 patients (5.3 lesions per participant on average), a 93% initial clearance after 3 months was observed.^4^ A subsequent prospective study with 12 patients (93 lesions) reported an initial clearance rate of 85% after 3 months.^5^

The primary objective of this study was to evaluate if SDL‐PDT was non‐inferior to C‐PDT in the treatment of AKs. Furthermore, the study aimed to evaluate pain during illumination, local skin reactions and patient preferences for both treatment methods.

## MATERIALS AND METHODS

### Study design and setting

This single‐centre, randomized intraindividual (split‐site) non‐inferiority trial evaluates the effectiveness of a single session with SDL‐PDT (experimental arm) versus C‐PDT (control arm) for treating mild to moderate AKs. In this set‐up, each participant received both treatments on the same anatomical site, with one half assigned to SDL‐PDT and the other to C‐PDT.

**FIGURE 1:**
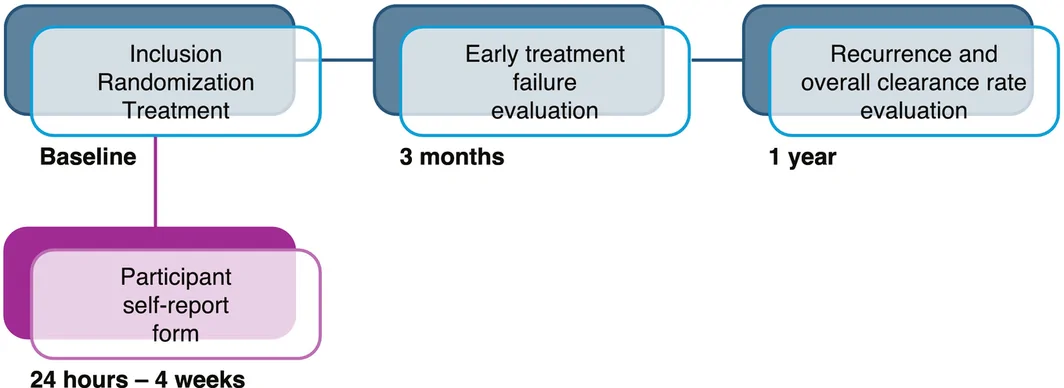
Overview of study visits and conducted procedures.

The study comprised a total of three visits, including one treatment session and two follow‐up appointments after 3 months (+/−2 weeks) and 1 year (+/−3 months) (Figure 1). The dataset encompasses all randomized patients with up to 1‐year follow‐up data compiled between February 2018 and September 2024. The findings are presented in accordance with the CONSORT 2010 statement^9^ and the TIDieR checklist, the template for intervention description and replication.^10^

### Ethics

The study followed the Declaration of Helsinki.^11^ The study received approval from the Regional Ethical Review Board in Gothenburg (initial approval 729‐17 and amendment T544‐18) and by the Swedish Ethical Review Authority (amendments 2020‐01775 and 2022‐01836‐02). All participants provided written informed consent. The trial was registered at http://www.researchweb.org/ (project number 264721).

### Participants

Participants were enrolled from the Department of Dermatology and Venereology at Sahlgrenska University Hospital, Gothenburg, Sweden. Eligible participants received study information and were scheduled for inclusion with a dermatologist after providing written informed consent. The eligibility criteria included adult patients with multiple, clinically diagnosed, mild to moderate AKs (Olsen grades I–II) suitable for PDT, symmetrically distributed on one or more anatomical sites. These sites included large areas that could be divided into two halves (e.g. scalp, forehead, torso) or bilateral body parts (e.g. cheeks, arms and/or dorsal hands). The exclusion criteria were patients who were unable to read or write Swedish, who could not comply with the treatment protocol, and those requiring nerve block anaesthesia in the treatment area during illumination. Data regarding participants' age and sex were collected as was the presence of immunosuppression (e.g. treatment with disease‐modifying anti‐rheumatic drugs, organ transplant recipient or chronic lymphocytic leukaemia).

### Interventions

The study employed a split‐site design. Ten dermatologists with extensive experience in diagnosing AK and performing PDT were responsible for patient and lesion inclusion while randomization and treatment administration were performed by two research nurses experienced in PDT.

All AKs were evaluated through clinical and dermoscopic examination using DermLite® DL200 Hybrid or DL4 dermoscopes (3Gen, San Juan Capistrano, CA, USA). Punch biopsies for histopathological confirmation were performed in case of diagnostic uncertainty. Clinical imaging was performed at inclusion and follow‐up visits. Prior to photography, AKs were outlined with a thin waterproof marker. A specified centreline was drawn, symmetrically dividing the lesions into two approximately equal groups. Each lesion situated to the left of the specified centreline was randomly assigned at the group level to receive either SDL‐PDT or C‐PDT. Conversely, lesions located on the right side received the alternative treatment. SDL‐PDT lesions were not occluded post‐prodrug application and were treated within 30 min, using simulated daylight (570–630 nm) from the Indoor Lux© illumination system (Gerdes Medical AG, Meckenheim, Germany) providing ≥12,000 lux for 2 h. For C‐PDT, lesions were occluded post‐prodrug application and incubated for 3 h followed by illumination with red light (635 ± 9 nm) from an Aktilite® CL 128 lamp (Galderma Holding SA, Lausanne, Switzerland) with a fluence of 37 J/cm^2^ over 8–9 min. SDL‐PDT was always performed first, using light‐proof coverings placed over the C‐PDT area. During C‐PDT illumination, SDL‐PDT treated areas were covered to prevent additional light exposure. All treatments were performed using the prodrug Ameluz® (Biofrontera Pharma GmbH, Leverkusen, Germany). Detailed treatment and evaluation procedures are described in Appendix S1.

### Objectives

The primary objective was to compare the clinical clearance rates after 1 year and to determine whether SDL‐PDT was non‐inferior to C‐PDT, allowing for an 8% difference in clearance rates. The secondary objectives were to assess pain and local skin reactions with both treatment methods as well as patient preferences.

### Outcomes

#### Primary outcome

The primary outcome was the overall clearance rate at 1 year defined as the percentage of all included AKs showing neither residual tumour (early treatment failure) at 3 months nor recurrence at 1 year.

#### Secondary outcomes

Patient‐reported pain during the illumination phase was measured using a numeric rating scale (NRS) ranging from 0 (‘no pain’) to 10 (‘worst possible pain’). The evaluation took place at the end of the illumination period as a summary of the pain experienced. After the treatment, participants were provided with a self‐report form (Appendix S2) to record local skin reactions, including redness, erosions, burning sensation, scaling and hyperpigmentation. Patients monitored these reactions at 24 h, 48 h, 7 days, 14 days and 28 days, using a severity scale from 0 to 3 (0 for none, 1 for mild, 2 for moderate and 3 for severe). Within this form, at the 24‐h mark, participants were also asked to respond to two additional questions regarding pain and their treatment preference.

### Randomization

A computer‐generated random allocation sequence with a block size of six was used to maintain balanced group sizes throughout enrolment. Randomization was conducted at the participant level; however, when a participant had lesions on distinct anatomical sites (e.g. forehead and arm), each site was randomized independently as a separate unit. To maintain confidentiality and minimize potential biases, the randomization process was documented in a printed Excel file, with access restricted solely to the research nurses. During the inclusion visit, lesions were identified by the dermatologist and divided into two treatment areas with an equal or nearly equal number of AKs. Blinding of the nurse and patient during treatment was not feasible, but the dermatologists remained unaware of the randomization at follow‐up visits.

### Statistical methods

#### Sample size

Prior to initiating the study, a power analysis was conducted to test the hypothesis of non‐inferiority assuming no true difference between C‐PDT (standard treatment) and SDL‐PDT (experimental treatment). Aiming for 95% statistical power and assuming a clearance rate of 80% with both methods, a non‐inferiority margin of 8% and a one‐sided 97.5% confidence interval (CI) at 1 year, the analysis determined that a total of 1300 lesions would be necessary (650 lesions per treatment group) to demonstrate that SDL‐PDT was non‐inferior to C‐PDT. Accounting for an expected dropout rate of 12.5%, our goal was to enrol 1463 lesions.

#### Statistical analysis

**FIGURE 2:**
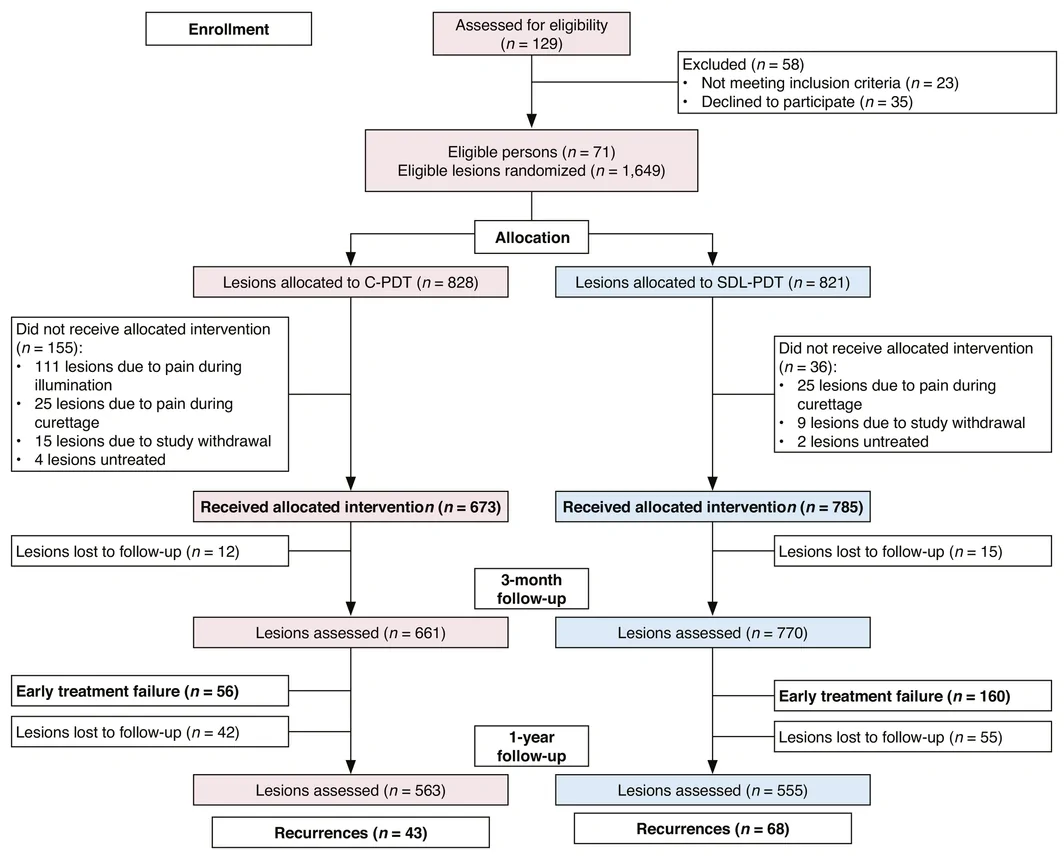
Study flow chart. C‐PDT, conventional photodynamic therapy; FU, follow‐up; *n*, numbers; SDL‐PDT, simulated daylight photodynamic therapy.

All data were analysed using R version 3.5.3 (The R Foundation for Statistical Computing, Vienna, Austria), except for the block randomization, which utilized version 3.0.3. Descriptive statistics, such as mean, median, range and quartiles, were utilized to characterize the data. Fisher's exact test was employed to compare proportions, while Wilcoxon's rank‐sum test was utilized for two‐sample comparisons. The modified intention‐to‐treat analysis on overall 1‐year clearance rates included all AKs receiving allocated treatment following randomization (Figure 2). Lesions with early treatment failure at 3 months or recurrence at 1 year were classified as non‐responders, while lesions lost to follow‐up were reported separately and were not imputed as failures. No sensitivity analyses for missing lesions were pre‐specified or performed. Analyses were performed at the lesion level because treatment allocation and clinical outcomes were defined per lesion within the split‐site design.

SDL‐PDT was considered non‐inferior if the lower bound of the CI did not exceed the prespecified margin of −8%. All analyses used two‐sided 95% CIs, except the non‐inferiority analysis, which used a one‐sided 97.5% CI.

## RESULTS

Overall, 129 participants were assessed for eligibility. After exclusions, 71 participants (mean age 74 [range 56–88] years; 71.6% men) with 1649 lesions were initially included. A total of 1458 AKs in 69 participants received the allocated treatment following randomization, with 673 lesions receiving C‐PDT and 785 lesions receiving SDL‐PDT. The total median number of lesions per participant was 21 (interquartile range, IQR: 14–28). The median number of lesions treated was 10 for both SDL‐PDT (IQR: 7–14) and for C‐PDT (IQR: 6.5–14.5). The majority of lesions were located on the face and scalp (89.9%), followed by other locations such as the chest, arms and dorsum of the hands (10.1%). Among the participants, 8.7% were immunosuppressed.

### Primary outcome

**FIGURE 3:**
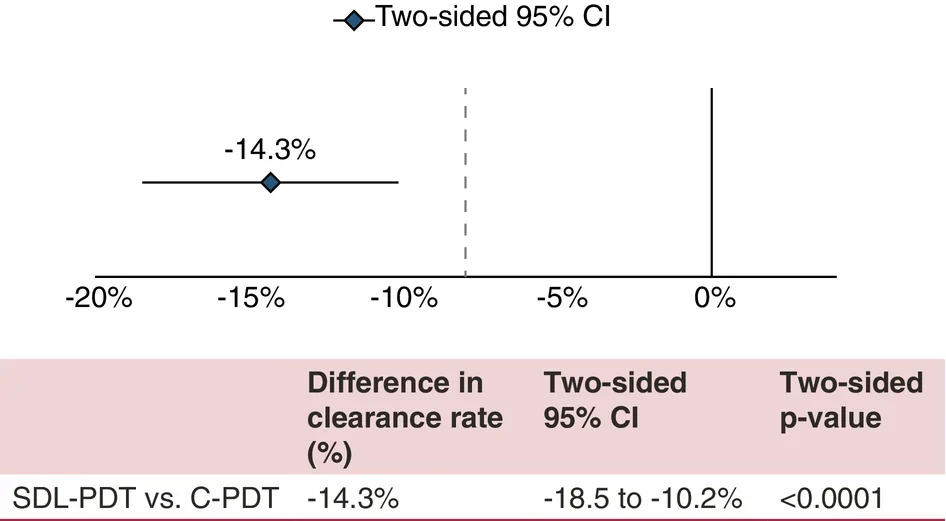
Non‐inferiority analysis showing lower effectiveness of simulated daylight photodynamic therapy compared to conventional photodynamic therapy 1 year after treatment, falling below the pre‐defined non‐inferiority margin of 8% (orange dotted line).

At 3 months, early treatment failure occurred in 160 lesions (20.8%) after SDL‐PDT and in 56 lesions (8.5%) after C‐PDT (*p* < 0.001). After 1 year, recurrence was observed for 68 lesions in the SDL‐PDT group and 43 lesions in the C‐PDT group. The overall clearance rate was 71.0% (95% CI, 67.6%–74.1%) for SDL‐PDT and 85.3% (95% CI, 82.4%–87.8%) for C‐PDT (*p* < 0.001) (Table 1). The non‐inferiority analysis confirmed that the absolute risk difference in lesion clearance between SDL‐PDT and C‐PDT was −14.3% (95% CI, −18.5% to −10.2%) (Figure 3).

**TABLE 1:** Overall clearance rates after 1 year.

Variables	C‐PDT	SDL‐PDT	*p*‐value
AK lesions, *n*	673	785	
1‐year clearance rate, *n* (%)	574 (85.3)	557 (71.0)	<0.001
95% CI	82.4–87.9	67.6–74.1	
Lost to follow‐up, *n*	54	70	

Abbreviations: CI, confidence interval; C‐PDT, conventional photodynamic therapy; *n*, number; SDL‐PDT, simulated daylight photodynamic therapy.

### Secondary outcomes

Participants reported more pain during illumination with C‐PDT (mean NRS score of 6.6) compared with SDL‐PDT (mean NRS score of 0.8) (*p* < 0.001). Water spray was required to relieve pain by 82.6% of patients during C‐PDT sessions. During SDL‐PDT sessions, water spray was not needed in any patients. Seven participants had to briefly pause C‐PDT illumination due to severe pain. However, they still completed the treatment, receiving the total light dose of 37 J/cm^2^. In the SDL‐PDT group, two participants paused the treatment for personal reasons (to use the restroom and pay for parking). During this time, the countdown was paused, and the treatment resumed until they had received the full 2 h of illumination (Table 2).

**TABLE 2:** Pain scores, use of water spray and pauses during illumination.

	C‐PDT	SDL‐PDT	*p*‐value
NRS pain score, mean (range)	6.7 (0–10)	0.8 (0–5)	<0.001
Use of water spray, *n* participants (%)	57 (82.6)	0 (0)	<0.001
Pauses, *n* participants (%)	14 (20.3)	2 (2.9)	0.0018

Abbreviations: C‐PDT, conventional photodynamic therapy; *n*, number; NRS, numeric rating scale; SDL‐PDT, simulated daylight photodynamic therapy.

The overall response rate for the self‐report forms among participants who received adequate illumination during SDL‐PDT and C‐PDT was 87.7% and 90.6%, respectively. Participants reported mild to moderate overall side effects. Burning sensation after 24 h was scored on average 0.9 for SDL‐PDT and 1.37 for C‐PDT on the 0–3 grading scale. Erosions and scaling after 1 week were present in both treatment groups, but symptoms were more pronounced in the C‐PDT group (average scores for erosion and scaling were 1.86 and 1.67, respectively) compared to the SDL‐PDT group (0.95 and 0.98, respectively). Among 60 respondents, 98.3% indicated a preference for SDL‐PDT 24 h after treatment.

### Adverse events

Beyond local skin reactions, no other adverse events could be linked to the study. Among 38 non‐related adverse events, 6 were serious (cervix cancer, colon cancer, disseminated melanoma disease, pyelonephritis, sepsis, transient ischaemic attack). There were no reports of suspected unexpected serious adverse reactions.

## DISCUSSION

This randomized controlled trial was a single‐centre, two‐armed, intraindividual (split‐site) study with a non‐inferiority design that evaluated the effectiveness, patient‐reported outcomes and local skin reactions of SDL‐PDT in comparison with C‐PDT in the treatment of mild to moderate AKs. While SDL‐PDT is inferior to C‐PDT in terms of 1‐year clearance rates, it is associated with significantly less pain and was preferred by patients immediately after treatment.

Direct comparisons between studies reporting on clearance rates should be made with caution, since variations in treatment protocols (including number of sessions, treatment intervals, type of photosensitizer, AK grade and localization) may influence results. Importantly, long‐term evaluations after a single treatment session with PDT are limited.

Previous studies on SDL‐PDT using ALA for mild to moderate AKs have reported clearance rates at 3 months similar to those observed here (85%–93%), but employed two treatment sessions, spaced 1 week apart.^4^, ^5^ To our knowledge, no other 1‐year follow‐up data on SDL‐PDT for AKs have been published, making this the first study to report such outcomes.

Regarding C‐PDT for AKs, one study evaluated the effectiveness of a single session of ALA C‐PDT showing a clearance rate of 93.7% after 3 months.^12^ Likewise, studies on C‐PDT using up to two sessions have shown lesion clearance rates at 3 months ranging from 81% to 94.3%.^13^, ^14^, ^15^, ^16^, ^17^, ^18^ Studies evaluating outcomes at 12 months with up to two C‐PDT sessions have shown clearance rates of 53.0%–77.5%.^19^, ^20^, ^21^ Interestingly, the 1‐year clearance rate with a single session of C‐PDT using ALA in this study (85.3%) was higher than that reported in prior studies. Although speculative, this may be attributable to high operator expertise, consistent treatment protocols and the use of the ALA formulation, which has been associated with higher effectiveness. These findings are consistent with our previous randomized controlled trial on SDL‐PDT versus C‐PDT for superficial basal cell carcinoma, which demonstrated similar effectiveness patterns with C‐PDT providing better clinical outcome than SDL‐PDT.^22^ Despite the fact that the specific ALA‐based single‐session SDL‐PDT protocol tested here is not directly comparable to previous studies using two sessions, SDL‐PDT may still hold value for highly pain‐sensitive patients despite lower effectiveness. Importantly, the comparison of two different interventions in this study highlights the importance of involving patients in treatment decisions. By providing clear information on effectiveness and patient‐reported outcomes, clinicians can support informed, person‐centred treatment choices, in line with recent European interdisciplinary AK guidelines emphasizing the role of PDT as an effective option.^23^

In this randomized controlled trial, pain during C‐PDT was significantly higher (mean NRS 6.6) compared to the minimal levels reported during SDL‐PDT illumination (mean NRS 0.8). Notably, 111 AKs randomized to C‐PDT were excluded because six patients interrupted treatment due to pain during illumination, whereas no patients in the SDL‐PDT group discontinued treatment for this reason. This disproportionate dropout may have introduced attrition bias, potentially affecting the observed pain levels and clearance rates for C‐PDT. However, the overall trends in pain and treatment preference remain clear, and the split‐site design minimized patient‐level confounding. The pain levels observed for C‐PDT are slightly higher than those reported in previous C‐PDT studies using ALA with mean pain visual analogue scale scores ranging from 4.1 to 5.8 out of 10 in two studies^14^, ^24^ and a median of 5.8 in another.^12^ This difference could be due to the lesion number per patient and total area treated with the prodrug. In our study, the mean number of lesions per participant on the C‐PDT‐treated side was 11.7, whereas the corresponding number in the above‐mentioned studies ranged from 6.1 to 6.9.^12^, ^14^ For SDL‐PDT, mean VAS pain scores of 0.1 were reported in two studies in which participants also had a lower mean number of treated lesions (ranging from 5.3 to 7.8 per participant).^4^, ^5^

Regarding participants' preference between C‐PDT and SDL‐PDT, a clear majority (98.3%) in the present study preferred SDL‐PDT when asked immediately after treatment. However, it is unclear whether their preference would have remained the same if they had been informed of the differences in clearance rates.

### Strengths and limitations

The strengths of this study include its randomized controlled design, a well‐defined protocol and an adequate power calculation. While the single‐centre design may limit generalizability, the rigorous methodology enhances robustness supporting broader applicability. Recurrences were assessed by dermoscopy rather than histopathology, which may have introduced some uncertainty; however, dermoscopy has demonstrated high sensitivity (95.6%) and specificity (95.0%) in diagnosing actinic keratoses.^25^ Loss to follow‐up was low and balanced between groups (8.0% vs. 8.9%), minimizing selection bias. Furthermore, the exact illumination dose with SDL‐PDT (lux) is less standardized compared to C‐PDT (J/cm^2^) and is difficult to determine. However, patient positioning followed a predefined protocol from a previous study by our group. This protocol ensures adequate illumination of the treatment area by positioning the patient between 0° and 45° relative to the ceiling light sources, while avoiding 90° angles.^3^ Pain was assessed at the end of illumination as a summary measure; therefore, peak pain during C‐PDT may have been higher than the reported summary scores. Although lesions were clustered within participants, the split‐site design enabled within‐participant comparisons, minimizing patient‐level confounding. Analyses were pre‐specified at the lesion level without planned patient‐level sensitivity analyses, which may limit assessment of clustering effects, although the split‐site design mitigates this. Clustering may have led to some underestimation of variance and overestimation of statistical precision, although conclusions are unlikely to be materially affected. Because both treatments were applied within the same participant, between‐participant variability was minimized. Another limitation of this study was the absence of long‐term follow‐up beyond the current 1‐year data. Additional follow‐up at 2 years will provide extended insights into the long‐term effectiveness of SDL‐PDT with ALA for mild to moderate AKs, and these data will be presented in a subsequent analysis.

## CONCLUSIONS

SDL‐PDT demonstrated a significantly lower clearance rate at 1 year than C‐PDT but was associated with significantly lower pain levels during illumination. The 1‐year follow‐up period provides valuable long‐term data; however, extended follow‐up is needed to assess later recurrences, and these data will be published in a future analysis.

## AUTHOR CONTRIBUTIONS

Conception or design of the work: AS, EB and JP. Data collection: All authors identified participants for eligibility. AS contacted all participants assessed for eligibility. MM, EB, JF, MC and JP included participants and lesions. AS treated the lesions. MM, EB, JF, MC and JP performed the evaluations at 3 and 12 months together with AS. MM, EB, JF, MC and JP evaluated the cosmetic outcome of treated lesions at 1 year. Data analysis and interpretation: AS, MC, EB and JP analysed the data. Drafting the article: AS drafted the article with support from MC and JP. Critical revision of the article: The article was revised by all authors. Final approval of the version to be published: All authors were responsible for the final approval, and only when they reached consensus was the article considered ready for submission.

## FUNDING INFORMATION

AS, EB, MM, JF and JP were supported by a grant from the Swedish state under the agreement between the Swedish government and the county councils, the ALF‐agreement (ALFGBG‐1006260). MC was supported by a research fellowship from the Swedish state under the agreement between the Swedish government and the county councils, the ALF‐agreement (ALFGBG‐942629).

## CONFLICT OF INTEREST STATEMENT

AS has acted as a consultant for Galenica AB, developing patient information leaflets and educational activities about photodynamic therapy directed to nurses and dermatology residents (2021–2025). EB, MM, JF, MC and JP have no conflicts of interest to declare.

## ETHICAL APPROVAL

The Regional Ethical Review Board in Gothenburg (Approval numbers: 743‐17; T544‐18) and the Swedish Ethical Review Authority (Approval numbers: 2020‐01775, 2022‐01836‐02) approved this study.

## ETHICS STATEMENT

The participants in this study gave written informed consent to the publication of their case details.

## Supporting information


Appendix S1.

Appendix S2.

## Data Availability

The data that support the findings of this study are available from the corresponding author upon reasonable request.
