# Supplementary material for: Simulated daylight versus conventional PDT for mild to moderate actinic keratoses: A randomized controlled trial
Source: J Eur Acad Dermatol Venereol. 2026 Jun 10;40(10):1713–20. doi: 10.1111/jdv.70556 (PMC13613350; doi:10.1111/jdv.70556)
Supplement: Supplementary file 1 [file JDV-40-1713-s001.docx]

**Appendix S2**

| Participant no. | | | | Initials | | | | Date of treatment | | | | | | |
| --- | --- | --- | --- | --- | --- | --- | --- | --- | --- | --- | --- | --- | --- | --- |
|  |  |  |  |  |  |  |  |  |  |  |  |  |  |  |

**Self-report form**

Instructions:

Fill in the self-report form after 24 hours, 48 hours, 7 days, 14 days and 28 days.

**After 24 hours**; DATE_____________

Mark the degree of side effects for the left and right side,

Respectively. The items are scored as follows:

0= None	1= Mild	2= Moderate	3= Severe

| After 24 hours | Left | Right |
| --- | --- | --- |
| Redness |  |  |
| Erosions |  |  |
| Burning sensation |  |  |
| Scaling |  |  |
| Hyperpigmentation |  |  |

**Did you experience less pain with one of the lamps?**

Yes ☐	No ☐

**If yes, which lamp did you prefer?**

White lamp in the room ☐ Red lamp ☐

**If you were to recive PDT again, which lamp would you prefer to be used?**

White lamp in the room ☐ Red lamp ☐ No preference☐

**After 48 hours**; DATE_____________

Mark the degree of side effects for the left and right side,

Respectively. The items are scored as follows:

0= None	1= Mild	2= Moderate	3= Severe

| After 48 hours | Left | Right |
| --- | --- | --- |
| Redness |  |  |
| Erosions |  |  |
| Burning sensation |  |  |
| Scaling |  |  |
| Hyperpigmentation |  |  |

**After 7 days**; DATE_____________

Mark the degree of side effects for the left and right side,

Respectively. The items are scored as follows:

0= None	1= Mild	2= Moderate	3= Severe

| After 7 days | Left | Right |
| --- | --- | --- |
| Redness |  |  |
| Erosions |  |  |
| Burning sensation |  |  |
| Scaling |  |  |
| Hyperpigmentation |  |  |

**After 14 days**; DATE_____________

Mark the degree of side effects for the left and right side,

Respectively. The items are scored as follows:

0= None	1= Mild	2= Moderate	3= Severe

| After 14 days | Left | Right |
| --- | --- | --- |
| Redness |  |  |
| Erosions |  |  |
| Burning sensation |  |  |
| Scaling |  |  |
| Hyperpigmentation |  |  |

**After 28 days**; DATE_____________

Mark the degree of side effects for the left and right side,

Respectively. The items are scored as follows:

0= None	1= Mild	2= Moderate	3= Severe

| After 28 days | Left | Right |
| --- | --- | --- |
| Redness |  |  |
| Erosions |  |  |
| Burning sensation |  |  |
| Scaling |  |  |
| Hyperpigmentation |  |  |
