# Supplementary material for: Simulated daylight versus conventional PDT for mild to moderate actinic keratoses: A randomized controlled trial
Source: J Eur Acad Dermatol Venereol. 2026 Jun 10;40(10):1713–20. doi: 10.1111/jdv.70556 (PMC13613350; doi:10.1111/jdv.70556)
Supplement: Supplementary file 2 [file JDV-40-1713-s002.docx]

**Supplemental Appendices**

**Appendix S1**

*Detailed treatment regimen*

Each treatment half or bilateral body part was randomly assigned to receive either simulated daylight photodynamic therapy (SDL-PDT) or conventional photodynamic therapy (C-PDT). Clinical photographs were taken before the treatment session. Prior to treatment initiation, treatment areas were cleansed using chlorhexidine alcohol (5 mg/ml), followed by gentle curettage to eliminate hyperkeratosis and enhance drug absorption. A disposable 7-mm dermal curette (Kai Medical, Tokyo, Japan) was used, with the blunt side applied to minimize bleeding while ensuring adequate exposure of the lesion surface. Curettage was performed in different directions, extending 10 mm into the surrounding healthy skin. If bleeding occurred, pressure was applied to the treatment area for at least 10 minutes. In rare cases, for lesions designated for C-PDT, an adrenaline-soaked gauze was briefly placed on the site for a few seconds. Once hemostasis was achieved, a topical gel containing BF-200 aminolevulinic acid (Ameluz®, Biofrontera Pharma GmbH, Leverkusen, Germany) was applied field-directed over the prepared areas.

For C-PDT, the treated area was covered with an occlusive, light-blocking dressing (Mediplast AB, Malmö, Sweden), along with four layers of aluminum foil (Quickpack Haushalt + Hygiene GmbH, Renningen, Germany), secured with surgical tape (Micropore™, Minnesota, USA). This dressing was applied within approximately 10 minutes of the gel application and remained in place for three hours before light exposure. In contrast, for SDL-PDT, no occlusion was used, and illumination began within 30 minutes of gel application, following European guidelines. [1]

C-PDT illumination was performed using an Aktilite® CL 128 lamp (Galderma Nordic AB, Uppsala, Sweden), emitting red light at 635 ± 9 nm with a total dose of 37 J/cm². The illumination lasted 8–9 minutes, depending on the lamp's specifications, with the light source positioned approximately 7 cm from the lesion (roughly the width of four fingers). SDL-PDT was administered using the IndoorLux© lighting system (Gerdes Medical AG, Meckenheim, Germany), which ensured a minimum illumination of 12,000 lux for two hours. The positioning of the treatment area followed established recommendations for optimal light exposure in an SDL-PDT setting. [2]

Both treatment areas were prepared simultaneously, with SDL-PDT illumination always conducted before C-PDT to optimize scheduling and minimize patient time at the clinic. During the C-PDT session, the SDL-PDT-treated area was shielded with a new light-proof dressing to prevent unintended light exposure. Following illumination, the treatment areas were cleansed with a damp paper towel.

After the treatment, participants received a self-report form (see Appendix S2) to document symptoms (such as redness, erosions, burning sensation, scaling, and hyperpigmentation), using a severity scale of 0-3 (0 for none, 1 for mild, 2 for moderate, and 3 for severe). Participants also answered three additional questions 24 hours post-treatment regarding perceived pain during illumination, and treatment preference. The questionnaire was completed at multiple time points: 24 hours, 48 hours, one week, two weeks, and four weeks post-treatment.

At the three-month follow-up visit, lesion healing was assessed by comparing photographs taken from inclusion. Primarily non-healed lesions in both treatment areas were identified, marked, photographed and documented. Additionally, areas with healed lesions were also photographed. At 12 months, all lesions were evaluated by comparing photos from inclusion and three-month follow-up. Treatment for the majority of lesions showing early treatment failure at 3 months or reccurence at 12 months were postponed until the 2-year evaluation. However, in the event of symptomatic problems, cryosurgery was offered.

[1]	Morton CA, Szeimies RM, Basset-Seguin N, Calzavara-Pinton P, Gilaberte Y, Haedersdal M, et al. European Dermatology Forum guidelines on topical photodynamic therapy 2019 Part 1: treatment delivery and established indications - actinic keratoses, Bowen's disease and basal cell carcinomas. J Eur Acad Dermatol Venereol. 2019;33; 2225-2238.

[2]	Sjöholm A, Claeson M, Paoli J. Measurements of illuminance in simulated daylight photodynamic therapy. Photodermatol Photoimmunol Photomed. 2022;38; 564-570.
